# HEPES‐buffering of bicarbonate‐containing culture medium perturbs lysosomal glucocerebrosidase activity

**DOI:** 10.1002/jcb.30234

**Published:** 2022-03-21

**Authors:** Martijn J. C. van der Lienden, Jan Aten, Rolf G. Boot, Marco van Eijk, Johannes M. F. G. Aerts, Chi‐Lin Kuo

**Affiliations:** ^1^ Department of Medical Biochemistry Leiden University Leiden The Netherlands; ^2^ Department of Pathology, Amsterdam UMC University of Amsterdam Amsterdam The Netherlands

**Keywords:** cell culture medium, diagnosis, Gaucher disease, glucocerebrosidase, glucosylsphingosine, lysosome

## Abstract

Glucocerebrosidase (GCase), encoded by the *GBA* gene, degrades the ubiquitous glycosphingolipid glucosylceramide. Inherited GCase deficiency causes Gaucher disease (GD). In addition, carriers of an abnormal *GBA* allele are at increased risk for Parkinson's disease. GCase undergoes extensive modification of its four *N*‐glycans en route to and inside the lysosome that is reflected in changes in molecular weight as detected with sodium dodecyl sulfate‐polyacrylamide gel electrophoresis. Fluorescent activity‐based probes (ABPs) that covalently label GCase in reaction‐based manner in vivo and in vitro allow sensitive visualization of GCase molecules. Using these ABPs, we studied the life cycle of GCase in cultured fibroblasts and macrophage‐like RAW264.7 cells. Specific attention was paid to the impact of 4‐(2‐hydroxyethyl)‐1‐piperazineethanesulfonic acid (HEPES) supplementation to bicarbonate‐buffered medium. Here, we report how HEPES‐buffered medium markedly influences processing of GCase, its lysosomal degradation, and the total cellular enzyme level. HEPES‐containing medium was also found to reduce maturation of other lysosomal enzymes (α‐glucosidase and β‐glucuronidase) in cells. The presence of HEPES in bicarbonate containing medium increases GCase activity in GD‐patient derived fibroblasts, illustrating how the supplementation of HEPES complicates the use of cultured cells for diagnosing GD.

## 1 INTRODUCTION

Glucocerebrosidase (GCase) is the lysosomal acid β‐glucosidase degrading glucosylceramide (GlcCer). Inherited defects in the *GBA* gene encoding GCase cause the lysosomal storage disorder Gaucher disease (GD).^1^, ^2^ More recently, mutations in *GBA* have been shown to pose a marked risk for developing Parkinson's disease and Lewy‐body dementia, even upon haploinsufficiency.^3^, ^4^ A hallmark of GD is lysosomal accumulation of GlcCer in tissue macrophages.^5^, ^6^ The lipid‐laden macrophages (Gaucher cells) are viable and contribute to the visceral GD symptoms, such as hepatosplenomegaly, thrombocytopenia, and anemia.^2^, ^5^ Most GD patients do not develop prominent complications in the central nervous system (CNS) and are designated as type 1. Nonneuronopathic type 1 GD is presently treated by macrophage targeted enzyme replacement therapy and substrate reduction therapy utilizing inhibitors of GlcCer biosynthesis.^6^, ^7^, ^8^, ^9^, ^10^, ^11^ Both approaches lead to impressive corrections in organomegaly and pancytopenia, which is preceded by corrections in plasma biomarkers of Gaucher cells.^12^

The availability of effective therapies has boosted laboratory diagnosis of GD, including (newborn) screening programs.^13^, ^14^ A step in GD diagnosis is demonstration of abnormalities in the GBA gene by sequencing. Demonstration of impaired GCase is performed by enzyme activity measurement, for which dried blood spots, white blood cells, and fibroblasts are used, depending on the laboratory. Unfortunately, neither genotyping nor the measurement of residual GCase activity in cell lysates accurately predicts onset and progression of GD in individual patients.^15^ Heteroallelic presence of the common N370S GBA mutation in GD patients is associated with absence of CNS involvement. The GBA genotype of GD patients does not always accurately predict severity of symptoms, even among siblings.^15^, ^16^ Monozygotic GD twins with different disease severity have even been documented.^17^, ^18^ Onset of GD disease can be sensitively detected by demonstration of elevated plasma protein markers of Gaucher cells, like chitotriosidase, C–C motif chemokine ligand 18, and glycoprotein nonmetastatic melanoma protein B, as well as elevated plasma glucosylsphingosine.^19^, ^20^, ^21^ Cell permeable fluorogenic substrates for in situ measurement of GCase activity in cultured cells have recently been developed.^22^, ^23^ Other recent tools to detect active GCase molecules in situ are fluorescent cyclophellitol‐based activity‐based probes (ABPs).^24^, ^25^ These cell permeable probes selectively react with GCase by covalent and irreversible binding to its catalytic nucleophile, E340. ABP‐labeled GCase molecules can be visualized by microscopy and gel electrophoresis.^24^, ^25^

**Figure 1:**
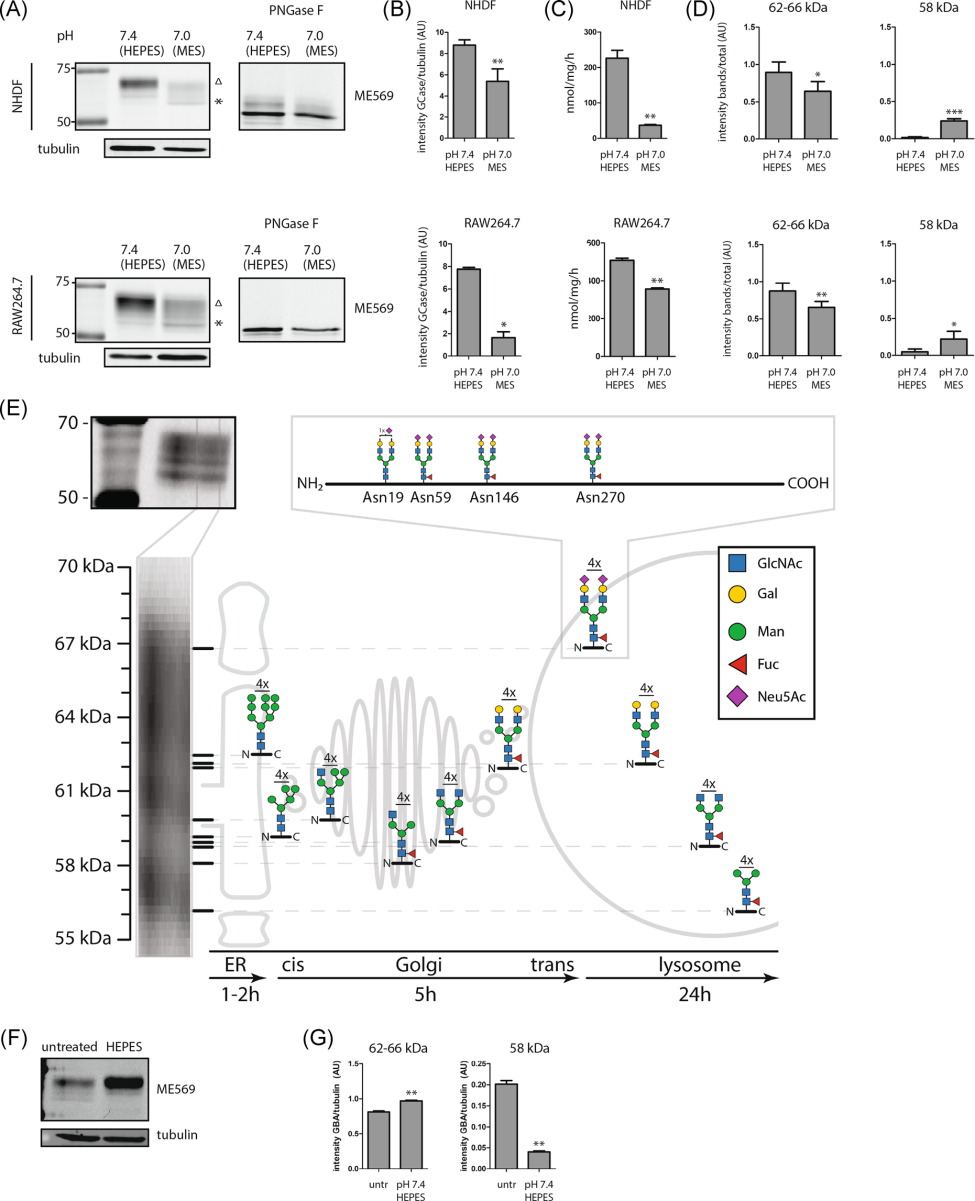
Impact of medium on cellular GCase glycoforms. (A) GCase in lysates of skin fibroblasts (NHDF) and RAW264.7 cells was labeled with GCase‐specific ABP and subsequently visualized by fluorescence scanning after SDS‐PAGE. Labeled GCase was digested with PNGase F to remove *N*‐glycans, as described in Section 2. (B) Quantification of total GCase band intensity shown in (A), corrected for tubulin loading control. (C) GCase of the same lysates of skin fibroblasts (NHDF) and RAW264.7 cells was measured with 4‐MU‐β‐Glc substrate as described in Section 2. (D) Quantification of prevalent GCase glycan isoforms shown in (A) defined as 62–66 and 58 kDa, as a proportion of total GCase intensity. (E) Scheme depicting processing of GCase glycoforms (adapted from Aerts, thesis). (F) Comparison of GCase glycan isoforms in lysates of cells cultured in bicarbonate buffered medium with and without supplementation with 50 mM HEPES. (G) Quantification of prevalent GCase glycan isoforms shown in (F) defined as 62–66 and 58 kDa, as a proportion of total GCase intensity. Significance (independent *t*‐test) is indicated by asterisks, **p ≤ 0.01. (A, E) ∆ indicates the 62–66 kDa isoforms, * indicates the 58 kDa isoform. 4‐MU‐β‐Glc, 4‐methylumbelliferyl substrate beta‐d‐glucopyranoside; ABP, activity‐based probe; ER, endoplasmic reticulum; HEPES, 4‐(2‐hydroxyethyl)‐1‐piperazineethanesulfonic acid; MES, 2‐(*N*‐morpholino)ethanesulfonic acid; NHDF, normal human dermal fibroblast; SDS‐PAGE, sodium dodecyl sulfate‐polyacrylamide gel electrophoresis

GCase is synthesized as 497 aa polypeptide containing four *N*‐linked glycans.^2^ The initially formed enzyme has a molecular weight (MW) of 62 kDa that subsequently increases to 66–69 kDa by modification of its glycans to sialylated complex type structures^26^ Inside the lysosomes, the local action of neuraminidase, β‐galactosidase, and β‐hexosaminidase cause stepwise reduction to the 58 kDa (“mature”) isoform (see also Figure 1E).^27^ Although the precise composition of the *N*‐glycans does not impact catalytic activity, *N*‐glycans are essential for correct folding of newly synthesized enzyme molecules in the endoplasmic reticulum (ER).^2^ Unlike most other lysosomal hydrolases, GCase in fibroblasts does not acquire mannose‐6‐phosphate moieties but is transported to lysosomes via binding to lysosomal integral membrane protein‐2 (LIMP‐2, encode by the *SCARB2* gene).^28^, ^29^, ^30^ The GCase/LIMP‐2 complex is sorted to lysosomes and dissociates upon low luminal pH.^31^ Pulse‐chase experiments in fibroblasts showed earlier that ^[35S]^methionine‐labeled GCase requires considerable time (several hours) to reach mature lysosomes, where it is relatively rapidly degraded by leupeptin‐sensitive proteases.^32^ GCase is already folded into active conformation in the ER, as can be detected with fluorogenic substrate and ABP labeling.^24^ Therefore, the measured total GCase activity in cell lysates does not necessarily reflect actual enzyme capacity in lysosomes.

Recently, we reported how 4‐(2‐hydroxyethyl)‐1‐piperazineethanesulfonic acid (HEPES)‐buffered medium impacts on lysosome maturation in cultured cells.^33^ Using GCase‐specific ABPs, we here demonstrate that cellular GCase is particularly influenced by such medium conditions. The presence of HEPES in the culture medium strikingly impairs maturation and reduces proteolytic turnover of GCase in lysosomes. This results in an apparent increase in total cellular enzyme level concomitant with relative absence in mature dense lysosomes.

## 2 MATERIALS AND METHODS

### 2.1 Cells and culture

RAW264.7 cells (American Type Culture Collection #TIB‐71) were cultured in Dulbecco's modified Eagle's medium (DMEM) and normal human dermal fibroblasts cells (NHDFs; Lonza #CC‐2511) were cultured in DMEM/F12. Both mediums contained 10% (vol/vol) fetal calf serum, 1% (wt/vol) glutamax and 0.2% (wt/vol) antibiotics (penicillin–streptomycin; all purchased from Thermo Fisher Scientific) at 37°C at 7% CO_2_ at controlled humidity. For modulation of medium pH, 2‐(*N*‐morpholino)ethanesulfonic acid (MES) (Sigma‐Aldrich; M3671), 3‐(*N*‐morpholino)propanesulfonic acid (MOPS) (Sigma‐Aldrich; M1254), and HEPES (Sigma‐Aldrich; H3375) were dissolved and filtered to obtain culture grade stock buffers (1 M). Where mentioned, culture medium was supplemented with culture grade HEPES, MES, or MOPS to a final concentration of 50 mM for at least 72 h, if not stated otherwise. Stock solutions were titrated so that final pH in medium was 7.0 for MES, 7.2 for MOPS, and 7.5 for HEPES. Leupeptin (Sigma‐Aldrich; L9783) was added in 25 or 50 µg/ml concentration to medium of cells pretreated with MES, MOPS, or HEPES for 72 h and incubated along with the respective buffers for 48 h. GD fibroblasts were obtained for fundamental investigations with consent of patients and their GBA genotype was confirmed by sequencing. The study was carried out in accordance with the Code of Ethics of the World Medical Association (Declaration of Helsinki).

### 2.2 ABP analysis

Cultured cells were lysed in KPi lysis buffer (25 mM K_2_HPO4/KH_2_PO4, pH 6.5, 0.1% [vol/vol] Triton X‐100) supplemented with protease inhibitors (Roche) and sonicated 5× 1 s with 9 s interval (amplitude 25%). Protein concentration was assessed by bicinchoninic acid assay (Thermo Fisher Scientific; 23225) and absorbance measurements (EMax Plus microplate reader; Molecular Devices). Equal protein amounts were labeled with excess of ABP conjugated to a fluorescent dye. Labeling of all active GCase molecules in cell homogenates was performed using 100 nM ABP‐ME569 (Cy5).^34^ Incubation was performed at 100 nM for 1 h (0.5%–1% [vol/vol] dimethylsulfoxide) on ice. Labeling of acid alpha‐glucosidase (GAA) and beta‐glucuronidase (GUSB) was performed as described earlier.^35^ Shortly, homogenates were prelabelled with 200 nM of β‐glc aziridine ABP JJB70 for 30 min at 37°C, pH 4.0 and 5.0, respectively. GAA was subsequently labeled by incubation of 500 nM JJB383 for 30 min at 37°C, pH 4.0. GUSB labeling was performed through incubation with 200 nM JJB392 for 30 min at 37°C, pH 5.0. After labeling, 5X Laemlli buffer (50% [vol/vol] 1 M Tris‐HCl, pH 6.8, 50% [vol/vol] 100% glycerol, 10% [wt/vol] dithiothreitol, 10% [wt/vol] sodium dodecyl sulfate [SDS], 0.01% [wt/vol] bromophenol blue) was added and samples were denatured at 95°C. Proteins were resolved by 10% polyacrylamide gel through SDS‐polyacrylamide gel electrophoresis (PAGE).

### 2.3 PNGase F treatment

Buffer exchange was performed on GCase‐labeled protein homogenate by spin desalting column (Pierce; 89849) and incubated with PNGase F according to the manufacturer's instructions (NEB; P0705S). Shortly, denaturation of protein was performed in denaturing buffer at 100°C for 10 min. Subsequent digestion by PNGase F was performed at 37°C for 1 h.

### 2.4 Pulse‐chase experiment

For in situ labeling of GCase in living cells, RAW264.7 cells, and NHDFs were cultured overnight in the presence of 100 nM green fluorescent cyclophellitol‐based ABP (MDW933).^24^ Next, cells were thoroughly washed and incubated with 100 nM red fluorescent ABP (MDW941)^24^ for different periods of times. Thus, existing GCase is labeled green and newly synthesized GCase is red. Cells were extensively washed, lysed in KPi lysis buffer, and equal amounts of protein were analyzed by SDS‐PAGE.

### 2.5 In‐gel visualization of probes

Detection of fluorescence in wet gel slabs was performed using a Typhoon FLA 9500 fluorescence scanner (GE Healthcare). Green fluorescence (MDW933 and JJB70) was detected using λ_EX_ 473 nm and λ_EM_ ≥ 510 nm, red fluorescence (MDW941) using λ_EX_ 532 nm and λ_EM_ ≥ 575 nm, and far‐red fluorescence (ABP‐ME569, JJB383, JJB392) using λ_EX_ 635 nm and λ_EM_ ≥ 665 nm.^35^ After imaging, gels were either stained by Coomassie G250 for total protein and scanned on ChemiDoc MP imager (Bio‐Rad; Figure S5) or used for western blotting.

### 2.6 Western blot analysis

Samples resolved on 10% polyacrylamide gels were transferred to 0.2 µm nitrocellulose membrane (#1704159; Biorad). Blocking of membranes occurred in 5% (wt/vol) bovine serum albumin (BSA; Sigma‐Aldrich; A1906) solution in phosphate‐buffered saline (PBS)/0.1% Tween‐20 (Sigma‐Aldrich; P1379) for 1 h at room temperature (RT). Primary antibodies against tubulin were from Cedarlane, CLT 9002 and secondary conjugated antibodies (Alexa Fluor^TM^ 488/647) from Molecular Probes. Scanning of immunoblots was performed using a Typhoon FLA 9500 fluorescence scanner (GE Healthcare).

### 2.7 Enzyme activity assays

Equal protein amounts as assessed by bicinchoninic acid assay were used for enzyme activity assays. GCase activity was assayed using 3.75 mM 4‐methylumbelliferyl (4‐MU) substrate beta‐d‐glucopyranoside (44059; Glycosynth) in McIlvaine buffer, pH 5.2, with 0.1% (wt/vol) BSA, 0.2% (wt/vol) sodium taurocholate, and 0.1% (vol/vol) Triton X‐100. For activity measurements of β‐hexosaminidases A/B, 5 mM 4‐MU‐β‐*N*‐acetyl‐glucosaminide (44007; Glycosynth) at pH 4.5 was used.

### 2.8 Density gradient fractionation

Cultured cells were harvested and washed 2X in PBS and 2X MME buffer (250 mM mannitol, 2 mM EGTA, 5 mM MOPS/Tris pH 7.0) through centrifugation at 1000 g for 5 min. Cells were resuspended in MME buffer and homogenized by 30 strokes using a Dounce homogenizer (B. Braun). The suspension was centrifuged for 2 min at 1000 rpm. The postnuclear fraction (supernatant) was transferred to a Percoll tube (49% Percoll [Sigma‐Aldrich; P1644], 250 mM mannitol, 2.5 mM MOPS‐Tris, and HCl titrated to pH 7.0) on top of a cushion of 2.5 M Sucrose (Sigma‐Aldrich). Ultracentrifugation of the column was performed at 30 000 g in a SW 41 Ti swinging bucket rotor (Beckman). Optimal density‐based fractionation was verified by Density Marker Beads (Pharmacia; 17‐0459‐01). After centrifugation, fractions of 250 µl were obtained and used for enzyme activity measurements.

### 2.9 Labeling of GCase in situ

Functionalized glass coverslips were seeded with NHDF at a confluency of 70% and treated with MES or HEPES. Active GCase was labeled by 2 h medium supplementation of 5 nM MDW941. Next, the cells were washed 3× with PBS and fixed with 4% (wt/vol) formaldehyde (Sigma‐Aldrich) in PBS for 20 min at RT while kept in the dark. Fixed cells were then washed with PBS and blocked in 5% normal donkey serum (NDS; Jackson Laboratory; 145‐017‐000‐121) for 60 min. Immunofluorescence staining was performed in 2% NDS. Rabbit anti‐LAMP‐1 (Abcam; AB24170) was used at a dilution of 1:400. Secondary antibody used was Alexa Fluor conjugated immunoglobulin G (H + L) donkey anti‐rabbit Alexa 488 (Invitrogen). Stained cells were mounted on a microscope slide with ProLong Diamond antifade reagent containing DAPI (Molecular Probes; P36962). Fluorescence microscopy was performed using a Leica TCS SP8 confocal microscope with a ×63/1.40 numerical aperture HC Plan Apo CS2 oil immersion objective and equipped with a hybrid detector.

### 2.10 Band quantification

Visualization of enzyme labeled bands in gel or western blot were quantified using automated image analysis software ImageQuant TL10.0 (Cytiva) and processed by Prism 8.0 (Graphpad Software).

### 2.11 Measurement of glucosylsphingosine

Levels of glucosylsphingosine in cultured fibroblasts were measured by a high‐performance liquid chromatography procedure, as earlier described.^36^

### 2.12 Statistical analysis

All experiments were independently performed at least twice. Significance was established upon a *p* ≤ 0.05 and indicated with asterisks. The standard deviation is depicted in the graphs as error bars.

## 3 RESULTS

### 3.1 Impact of medium pH on cellular GCase glycoforms

Murine macrophage‐like RAW264.7 cells and human skin fibroblasts were cultured in DMEM and DMEM/F12 medium, respectively, at 7% CO_2_. Different buffers were added to the medium at a final concentration of 50 mM: MES (pKa = 6.15) or HEPES (pKa = 7.5). The final medium pH was 7.0 and 7.4, respectively. After a week, cells were harvested and lysed in KPi‐buffer supplemented with 0.1% Triton X‐100. Active GCase molecules in cell lysates were labeled with fluorescent ABP ME569 and were analyzed by SDS‐PAGE and fluorescence scanning.

The cellular GCase content and glycoform profile was found to be clearly influenced by the medium composition. In fibroblasts and RAW264.7 cells cultured at lower pH with MES, relatively little labeled GCase was present (Figure 1A, quantified in Figure 1B), and relatively little GCase activity was detected (Figure 1C). In cells cultured at pH 7.4, in the presence of HEPES, GCase activity and protein was more abundant, in particular, glycoforms with MW of 62–66 kDa (Figure 1A, quantified in Figure 1B,D). PNGase digestion resulted in the generation of a 52 kDa labeled protein in lysates of both MES and HEPES exposed cells, which confirms that all labeled enzyme is GCase and indicates that the various MW forms stem from differences in glycan composition (Figure 1A,E). Of note, clear enrichment of the high MW variants was observed upon 72 h HEPES supplementation compared with bicarbonate buffered medium alone (Figure 1F,G). A similar shift in glycan isoforms was observed upon stimulation with 25 mM HEPES, a concentration used in commercially available culture medium (Figure S1A). We also studied cells that were exposed to 50 mM MOPS; pKa = 7.15), buffering the medium pH at 7.15. As shown in Figure S1B, cells cultured at pH 7.15 showed an intermediate GCase profile when compared to that of cells cultured at higher and lower medium pH.

### 3.2 Dynamics of induced changes in GCase by HEPES‐buffered medium

**Figure 2:**
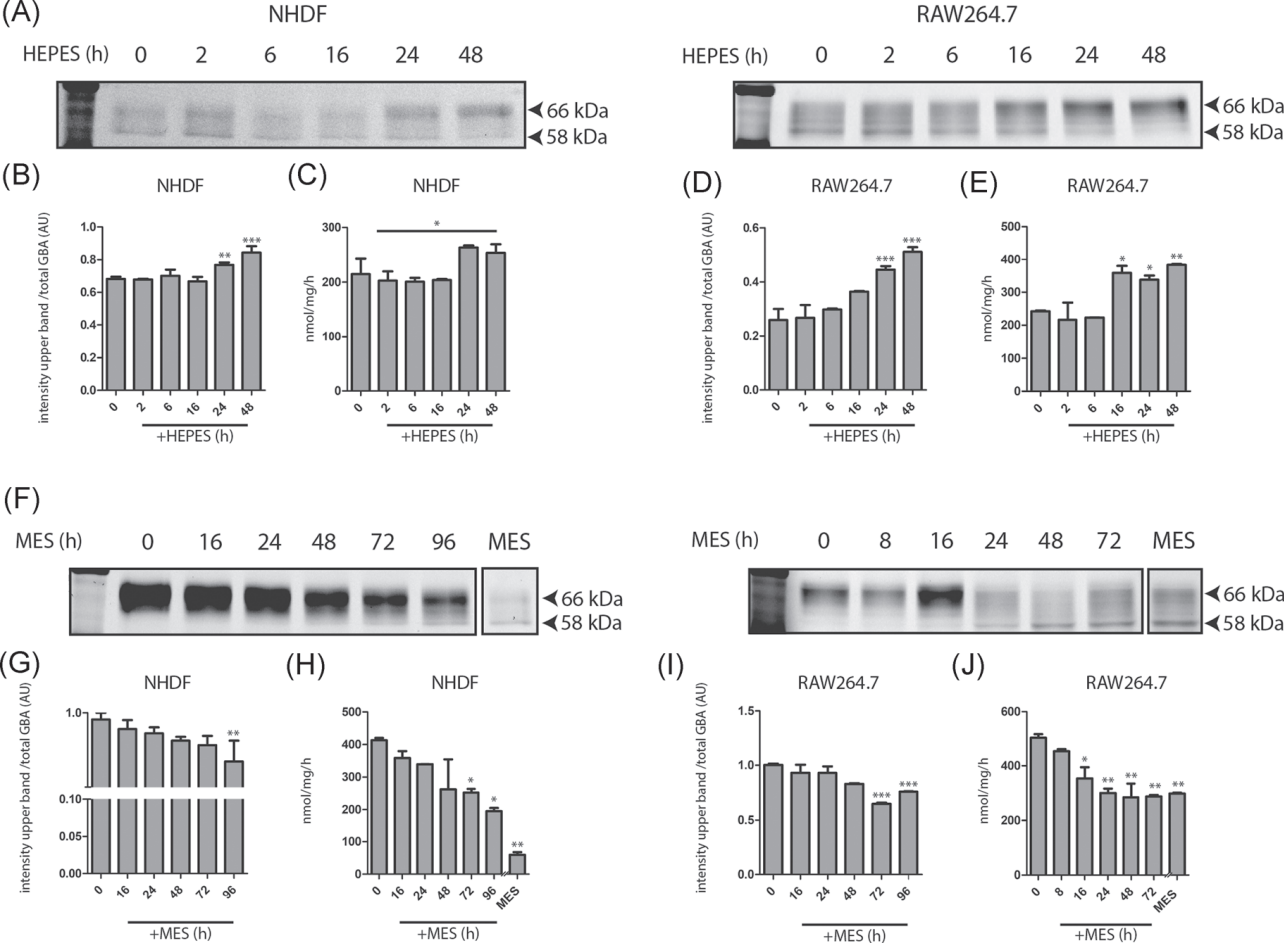
Induction and reversibility of GCase changes by HEPES‐containing medium. (A) Induction. Skin fibroblasts (NHDF) and RAW264.7 cells were exposed to either 50 mM HEPES or MES, and cellular GCase was monitored in time (0–48 h) by means of ABP labeling of enzyme in cell lysates and the measurement of enzymatic activity in lysates. (B) Quantified intensity of GCase glycan isoforms 62–66 in NHDF lysates depicted in (A), corrected for total GCase (total). (C) GCase activity of the same lysates of NHDFs was measured with 4‐MU‐β‐Glc substrate as described in Section 2. (D) Quantified intensity of GCase glycan isoforms 62‐66 in RAW264.7 lysates depicted in (A), corrected for total GCase (total). (E) GCase activity of the same lysates of RAW264.7 was measured with 4‐MU‐β‐Glc substrate as described in Section 2. (F) Reversibility. Skin fibroblasts (NHDF) and RAW264.7 cells were exposed for 3 days to 50 mM HEPES in the culture medium (pH 7.4). Following washing, cells were cultured in medium containing 50 mM MES (medium pH 7.0), and cellular GCase was monitored in time (0–96 h) in cell lysates by means of ABP labeling of enzyme molecules and measurement of GCase activity. (G) Quantified intensity of GCase glycan isoforms 62–66 in NHDF lysates depicted in (F), corrected for total GCase (total). (H) GCase activity of the same lysates of NHDFs was measured with 4‐MU‐β‐Glc substrate as described in Section 2. (I) Quantified intensity of GCase glycan isoforms 62–66 kDa in RAW264.7 lysates depicted in (F), corrected for total GCase (total). (J) GCase activity of the same lysates of RAW264.7 was measured with 4‐MU‐β‐Glc substrate as described in Section 2. The last lane to the right represents cells chronically cultured in in the presence of 50 mM MES. Overall significance of treatment effect (one‐way ANOVA, Tukey post hoc) is indicated by graph‐wide asterisks, individual asterisks on bars indicate significance compared to *t* = 0 (A) or HEPES (B). *p ≤ 0.05, **p ≤ 0.01, ***p ≤ 0.001. 4‐MU‐β‐Glc, 4‐methylumbelliferyl substrate beta‐d‐glucopyranoside; ABP, activity‐based probe; ANOVA, analysis of variance; HEPES, 4‐(2‐hydroxyethyl)‐1‐piperazineethanesulfonic acid; MES, 2‐(*N*‐morpholino)ethanesulfonic acid; NHDF, normal human dermal fibroblast

The induction and reversibility of changes in cellular GCase induced by culture medium were investigated more closely. For this, cells (fibroblasts and RAW264.7 cells) grown in culture medium buffered by bicarbonate were exposed to culture medium supplemented with 50 mM HEPES (medium pH 7.4). In both cell types, GCase with higher MW, reflecting more sialylated complex glycans, accumulated (Figure 2A, quantified in Figure 2B,D) and overall GCase activity increased over time (Figure 2C,E). Next, the reversibility of the induced changes in GCase was examined. Cells were first exposed to medium containing 50 mM HEPES for 3 days. Subsequently, cells were washed and further cultured in the presence of 50 mM MES (medium pH 7.0). At different time points (0–96 h), cells were harvested and cellular GCase was studied by ABP‐labeling and SDS‐PAGE, as well as by enzymatic activity measurements (Figure 2F–J). Chase at lower medium pH caused a reversal of the GCase glycoform profile (Figure 2F, quantified in Figure 2G,I), which was accompanied by reduced total cellular enzymatic activity (Figure 2H,J). Of note, both the induction of altered glycoform composition and the correction of GCase proceeded slower in fibroblasts compared to RAW264.7 cells (Figure 2).

### 3.3 Life cycle of GCase visualized with ABPs

**Figure 3:**
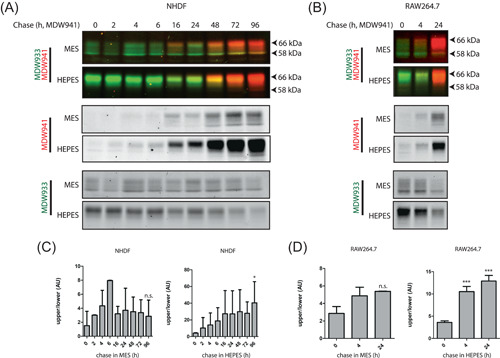
Visualization of GCase isoforms with two distinct ABPs: reduced glycan maturation in HEPES‐containing medium. (A) Pulse‐chase experiments with fibroblasts, performed as described in Section 2. Following prelabeling with MDW933 (green fluorescent, existing GCase), cells were incubated continuously with MDW941 (red fluorescent, newly synthesized GCase) for indicated time periods. Cells were harvested and labeled GCase was visualized following SDS‐PAGE. (B) Same experimental setup was used for GCase studies in RAW264.7 cells. (C) Quantification of band intensity shown in (A) depicting the ratio of newly formed GCase (MDW941) glycan isoforms 62–66 (upper) over 58 (lower) kDa in MES (left) and HEPES (right). (D) Quantification of band intensity shown in (B) depicting the ratio of newly formed GCase (MDW941) glycan isoforms 62–66 (upper) over 58 (lower) kDa in MES (left) and HEPES (right). Overall significance of treatment effect (one‐way ANOVA, Tukey post hoc) is indicated by graph‐wide asterisks, individual asterisks on bars indicate significance compared to *t* = 0 (A) or HEPES (B); *p ≤ 0.05, **p ≤ 0.01, ***p ≤ 0.001. ABP, activity‐based probe; ANOVA, analysis of variance; HEPES, 4‐(2‐hydroxyethyl)‐1‐piperazineethanesulfonic acid; MES, 2‐(*N*‐morpholino)ethanesulfonic acid; NHDF, normal human dermal fibroblast; SDS‐PAGE, sodium dodecyl sulfate‐polyacrylamide gel electrophoresis

Two GCase‐specific ABPs conjugated with green and red boron dipyrromethene, MDW933 and MDW941, respectively,^24^ were used to perform a pulse‐chase experiment. Cultured fibroblasts and RAW264.7 cells were first exposed to 100 nM MDW933 overnight to irreversibly label all active GCase molecules. Next, cells were extensively washed and subsequently cultured in the presence of red fluorescent MDW941. Detection of MDW941‐labeled enzyme allows selective monitoring of de novo synthesized GCase in time. The pulse‐chase experiments were performed with cells cultured in medium containing either 50 mM HEPES or 50 mM MES. Cells were harvested at different time points during the chase period (0–96 h) and cellular GCase was analyzed by SDS‐PAGE (Figure 3). Incubation of fibroblasts and RAW264.7 cells with the red MDW941 probe for extended periods of time resulted in complete labeling of GCase and its complete inactivation as measured with the activity assay (Figure 3, Figure S2). During the chase period, MDW941‐labeled GCase increased in both cell types cultured in both media, indicating sustained synthesis of GCase during the various chases (Figure 3A,B). Cells cultured with HEPES did not show the transition of 66 kDa GCase to 58 kDa enzyme, a process known to depend on stepwise removal of external sugars from the N‐glycans (Figure 3A,B).^27^ In contrast, cells cultured with 50 mM MES at pH 7.0 did show detectable formation of 58 kDa GCase after one day of chase, and all bands remain present during the chase (Figure 3). This is reflected in the quantification of the separate bands (Figure 3C,D), as the ratio of the upper band compared to the lower band intensity remains the same when MES is present, whereas enrichment of the upper band occurs in the presence of HEPES in both cell types. Again, the generation of mature GCase occurred slower in NHDF compared to RAW264.7 cells.

### 3.4 Subcellular localization of GCase in cells cultured in the presence of different buffers

**Figure 4:**
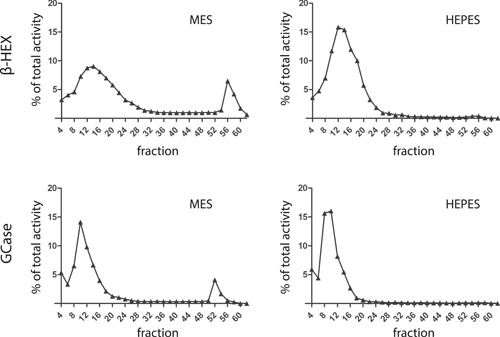
Subcellular fractionation of fibroblasts cultured in the presence of 50 mM HEPES or MES. Homogenates of RAW264.7 cells treated with HEPES or MES were fractionated and compartments were separated on the basis of density using 49% Percoll centrifugation to generate density gradients. In collected fractions, enzymatic activities of GCase and β‐hexosaminidase were measured as described in Section 2. HEPES, 4‐(2‐hydroxyethyl)‐1‐piperazineethanesulfonic acid; MES, 2‐(*N*‐morpholino)ethanesulfonic acid

The GCase activity levels in cultured fibroblast cell lines are notoriously variable,^37^ tending to be lower in cells exposed to more acid medium when being more confluent. To identify the impact of medium conditions we deliberately made the comparison between HEPES and MES buffered cells. Subcellular fractionation was used to separate compartments by a continuous Percoll density gradient, as described in Section 2. In gradient fractions, the enzyme activities of GCase and the lysosomal enzyme β‐hexosaminidase were determined. In the case of MES‐exposed RAW264.7 cells, GCase and β‐hexosaminidase activities were detected in fractions with high density, known to contain mature dense lysosomes (Figure 4). GCase and β‐hexosaminidase activity in cells exposed to HEPES was virtually absent in dense fractions. The lower density fractions contain prelysosomal compartments (ER, Golgi, endolysosomes, and immature lysosomes). HEPES‐exposed cells show relatively high levels of lysosomal enzymes in these fractions (Figure 4). Similar observations were made for fibroblasts (Figure S3A). Notably, labeled GCase remained colocalized with LAMP1 positive vesicles in HEPES cultured conditions (Figure S3B) Thus, HEPES‐exposed cells contain on average less GCase and β‐hexosaminidase in dense endolysosomes as compared to MES‐exposed cells.

**Figure 5:**
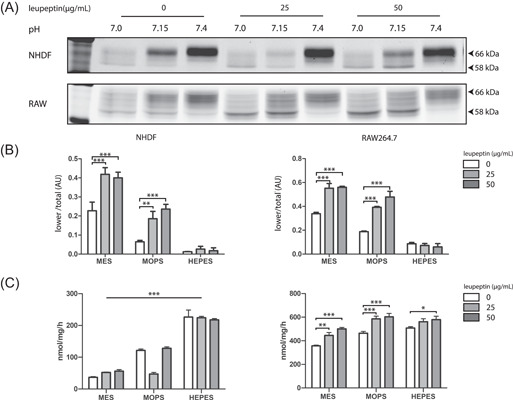
Inhibition of lysosomal cathepsins increases GCase in cells exposed to MES and MOPS, but not those exposed to HEPES. Cells (fibroblasts and RAW264.7) were cultured in the presence of 50 mM buffer compound (MES, MOPS, or HEPES) in the absence or presence of 0, 25, or 50 µg/ml leupeptin for 48 h. Cells were harvested and GCase in lysates was visualized by (A) ABP labeling, SDS‐PAGE, and fluorescence scanning. (B) Quantified intensity of GCase glycan isoforms 58 kDa of bands depicted in (A), corrected for total GCase (total). (C) Enzymatic GCase activity measurements (as described in Section 2) of same lysates. Overall significance of interaction (two‐way ANOVA) is indicated by graph‐wide asterisks, individual asterisks on bars indicate significance (Bonferroni post hoc) compared to 0 µg/ml leupeptin, *p ≤ 0.05, **p ≤ 0.01, ***p ≤ 0.001. ABP, activity‐based probe; ANOVA, analysis of variance; HEPES, 4‐(2‐hydroxyethyl)‐1‐piperazineethanesulfonic acid; MES, 2‐(*N*‐morpholino)ethanesulfonic acid; MOPS, 3‐(*N*‐morpholino)propanesulfonic acid; NHDF, normal human dermal fibroblast; SDS‐PAGE, sodium dodecyl sulfate‐polyacrylamide gel electrophoresis

Lysosomal proteolytic degradation of GCase is potently inhibited by leupeptin, a broad protease inhibitor.^36^, ^38^ Consequently, leupeptin induces accumulation of mature 58 kDa GCase. Overall, the presence of leupeptin led to accumulation of 58 kDa GCase (Figure 5A, quantified in Figure 5B) and to an increase of GCase activity (Figure 5C) in cells cultured with MES or MOPS but not in those cultured with HEPES. Apparently, GCase in HEPES‐treated cells hardly reaches dense endolysosomes where proteolytic degradation occurs.

### 3.5 Implications for diagnosis of GD using cultured cells

**Figure 6:**
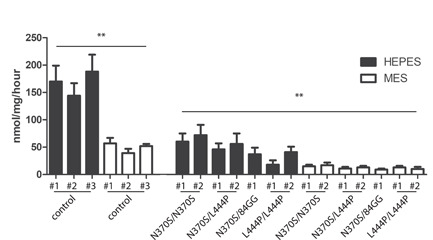
GCase activity level in control and Gaucher fibroblasts cultured in the presence of 25 mM MES or HEPES. Fibroblast obtained from healthy individuals and Gaucher patients with known *GBA* genotype were cultured as indicated and GCase activity in cell lysates was determined. Values expressed a mean ± SD; measurements performed in triplicate. Significance (paired *t*‐test, asterisks) indicates the effect of buffer on control and GD‐patient cells, respectively; *p ≤ 0.05, **p ≤ 0.01, ***p ≤ 0.001. Hash (#) indicates cell lines derived from patients harboring the same mutation. GD, Gaucher disease; HEPES, 4‐(2‐hydroxyethyl)‐1‐piperazineethanesulfonic acid; MES, 2‐(*N*‐morpholino)ethanesulfonic acid

The use of culture medium containing HEPES is increasingly popular because it ensures stable buffering of medium for several days.^39^ Cellular GCase with a relative short lysosomal life span appears particularly influenced by the use of HEPES buffer in the culture medium, a phenomenon with important repercussions for GD diagnosis. Figure 6 shows the GCase levels (nmol/mg protein/hour) in lysates of fibroblasts from type 1 GD patients and normal individuals cultured in the presence of HEPES or MES. The enzyme levels in lysates of patient cells cultured in the presence of HEPES in some cases overlap with those in lysates of cells from normal individuals cultured in the presence of MES. Thus, culturing patient and control cells at different medium conditions might result in false negatives in GD diagnosis. Consistently, glucosylsphingosine levels in patient‐derived fibroblasts were slightly increased when cells were cultured in the presence of 25 mM HEPES. Since glucosylsphingosine is the most potently increased lipid upon GCase‐deficiency,^40^, ^41^ this finding points again to reduced lysosomal GCase activity in lysosomes of living cells, as opposed to the elevated GCase activity detected in lysates using the 4‐MU‐substrate activity assay (Table 1).

**Table 1:** Glucosylsphingosine (GlcSph) content of fibroblasts cultured in the absence or presence of 25 mM HEPES

Fibroblast line	No HEPES	+25 mM HEPES (pH 7.4)
	GlcSph (pmol/mg total protein of cell lysate)	
Control wt GBA	<0.3	<0.3
Control wt GBA	<0.3	<0.3
N370S/L444P GBA	1.3 ± 0.4	4.2 ± 1.2
N370S/L444P GBA	0.9 ± 0.3	3.3 ± 0.6
N370S/N370S GBA	0.7 ± 0.3	1.4 ± 0.5
L444P/L444P GBA	2.5 ± 0.4	2.4 ± 0.8

*Note*: Values expressed as mean ± SD on three independent cell cultures and triplicate measurements.

Abbreviation: HEPES, 4‐(2‐hydroxyethyl)‐1‐piperazineethanesulfonic acid.

### 3.6 Impact of medium on other lysosomal glycosidases

**Figure 7:**
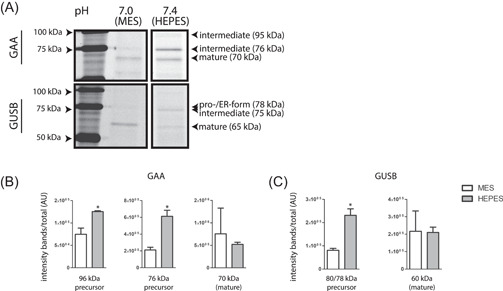
Impact of medium pH on acid alpha‐glucosidase (GAA) and beta‐glucuronidase (GUSB) isoforms. (A) Fibroblasts were cultured in the presence of 50 mM buffer compound (MES or HEPES). Cells were harvested and GAA and GUSB in lysates was visualized by ABP labeling, SDS‐PAGE, and fluorescence scanning. (B) Quantified intensity of GAA isoforms depicted in (A), corrected for total GAA intensity. (C) Quantified intensity of GUSB isoforms depicted in (A), corrected for total GUSB intensity. ABP, activity‐based probes; HEPES, 4‐(2‐hydroxyethyl)‐1‐piperazineethanesulfonic acid; MES, 2‐(*N*‐morpholino)ethanesulfonic acid; SDS‐PAGE, sodium dodecyl sulfate‐polyacrylamide gel electrophoresis

Selective ABPs have become available for a number of other lysosomal retaining glycosidases like acid GAA and GUSB.^38^, ^42^ We examined the impact of the culture medium buffers on these enzymes using corresponding ABPs for visualization. Figure 7 shows that in fibroblasts cultured in the presence of HEPES at a medium pH of 7.4, the ratio of intermediate and mature GAA is altered, pointing to perturbed maturation (quantified in Figure 7B). Likewise, an increase in the intermediate form of GUSB (75 kDa) and a decrease in the mature form (65 kDa) was noted in cells cultured in the presence of HEPES (Figure 7A, quantified in Figure 7C). Proteolytic processing of 95 and 76 kDa GAA and 75 kDa GUSB is thought to largely take place in lysosomes. The findings therefore suggest that the involved proteases in this processing are less active. This explanation was substantiated by the finding that leupeptin treatment did not cause an increase in mature 65 kDa GUSB in fibroblasts cultured in the presence of HEPES (Figure S4). Of note, the intermediate 75 kDa GUSB was increased in cells cultured in the presence of HEPES and leupeptin (Figure S4).

## 4 DISCUSSION

Many investigations on GCase make use of cultured cells. Earlier work in our lab revealed that culture conditions may impact on autophagy and lysosomes in cells, in particular the popular addition of HEPES to culture medium that maintains a relatively high medium pH of 7.4.^33^ A more recent study by Cook et al.^43^ demonstrated reduction of lysosomal calcium concentration by the exposure to the buffer. Our present investigation illustrates the marked influence of the presence of HEPES in the culture medium on cellular GCase, both qualitatively and quantitatively. In cells, fibroblasts and macrophage‐like RAW264.7 cells alike, exposure to HEPES containing medium causes GCase to steadily accumulate. The accumulating enzyme shows a MW of about 66 kDa, which suggests an abundance of complex‐type sialylated glycans. The subsequent intralysosomal conversion to a 58 kDa glycoform by trimming of *N*‐glycans is less apparent in cells that are exposed to HEPES. The observed reduction in GCase glycan processing might theoretically be caused by an arrest of the enzyme in the trans‐Golgi region in cells exposed to HEPES. However, it seems more likely that mature lysosomes acquire a higher pH upon uptake of HEPES, consequently exhibit lower density and have reduced hydrolase capacities.^33^ Other explanations cannot be entirely excluded yet. For example, the relatively high medium pH might impact on cytosolic pH, which, in turn, could influence lysosome acidification via STAT3.^44^ The effects of the presence of HEPES in the medium on cellular GCase were more prominent in RAW cells than fibroblasts. This difference might be due to more prominent endocytotic uptake and delivery to lysosomes of HEPES by RAW264.7 cells when the cells are exposed a few days to the buffer containing medium.

## 5 CONCLUSION

Our study revealed that the presence of HEPES in the cell culture medium significantly impacts on cellular GCase. The enzyme is less present in dense mature lysosomes and might therefore be relatively inactive towards substrate in vivo. Indeed, we noted that formation of glucosylsphingosine in lysosomes, an indirect measure for impaired degradation of GlcCer,^40^ is significantly higher in type 1 GD fibroblasts when cultured in the presence of HEPES (Table 1). When employing cultured cells for GD diagnosis, the use of medium additives such as HEPES is not advisable. This popular buffer causes an artificial accumulation of GCase in cells that might disturb accurate diagnosis. Other lysosomal enzymes (GAA and GUSB) also appear to mature slower in cells exposed to HEPES‐buffered medium.

## AUTHOR CONTRIBUTIONS

**Martijn J. C. van der Lienden**: Investigation and writing. **Jan Aten**: Investigation: **Rolf G. Boot**: Verification. **Marco van Eijk**: Supervision and writing. **Johannes M. F. G. Aerts**: Conceptualization and writing. **Chin‐Lin Kuo**: Conceptualization and writing.

## CONFLICTS OF INTEREST

The authors declare no conflicts of interest.

## Supporting information

Supporting information.

## Data Availability

Data are available from the corresponding author upon reasonable request.
