# Supplementary material for: HEPES‐buffering of bicarbonate‐containing culture medium perturbs lysosomal glucocerebrosidase activity
Source: J Cell Biochem. 2022 Mar 21;123(5):893–905. doi: 10.1002/jcb.30234 (PMC9314694; doi:10.1002/jcb.30234)
Supplement: Supplementary file 1 — Supporting information. [file JCB-123-893-s001.docx]

**Supplemental figures**

**
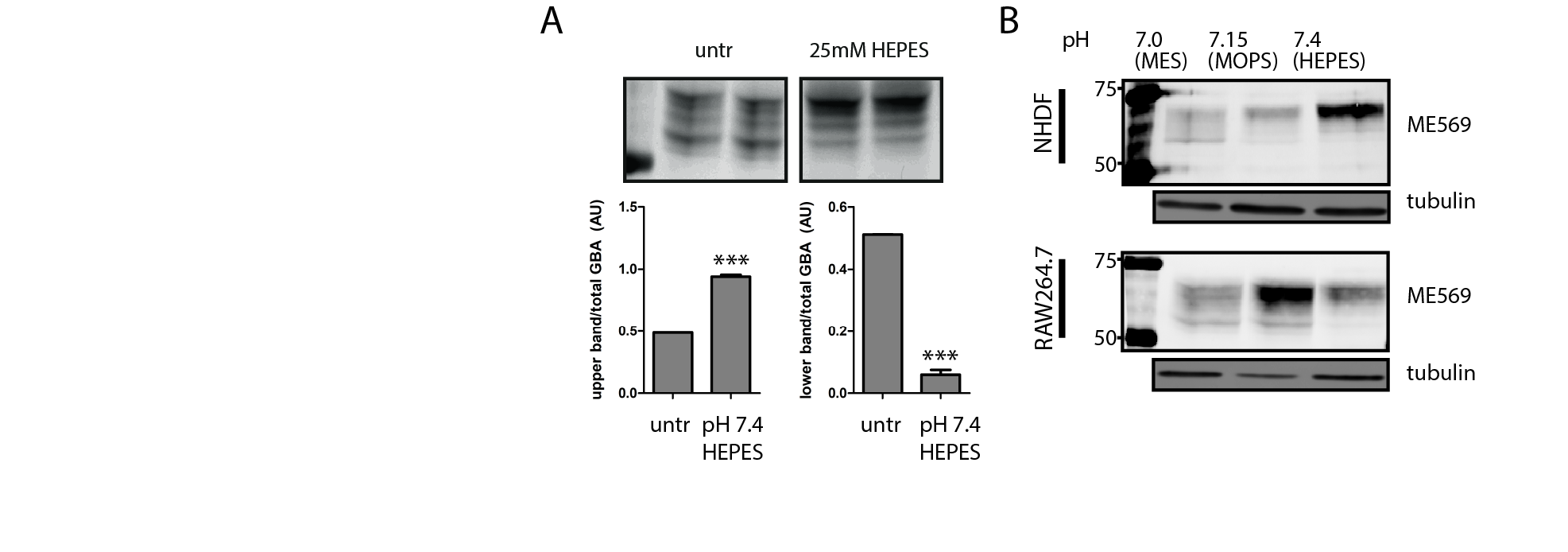
**

**Figure S1. Impact of the presence of zwitterionic buffers in medium on GCase in cultured fibroblasts and RAW264.7 cells.** **(A)** Comparison of GCase glycan isoforms in lysates of cells cultured in bicarbonate buffered medium with and without supplementation with 25 mM HEPES and quantification (lower panel) of upper bands (62-66kDa) and lower bands (58kDa) **(B)** GCase in lysates derived from 50mM MES, MOPS and HEPES exposed cells was labelled with GCase-specific ABP and subsequently visualized by fluorescence scanning after SDS-PAGE. Significance in (A) is indicated by asterisks (independent t-test).


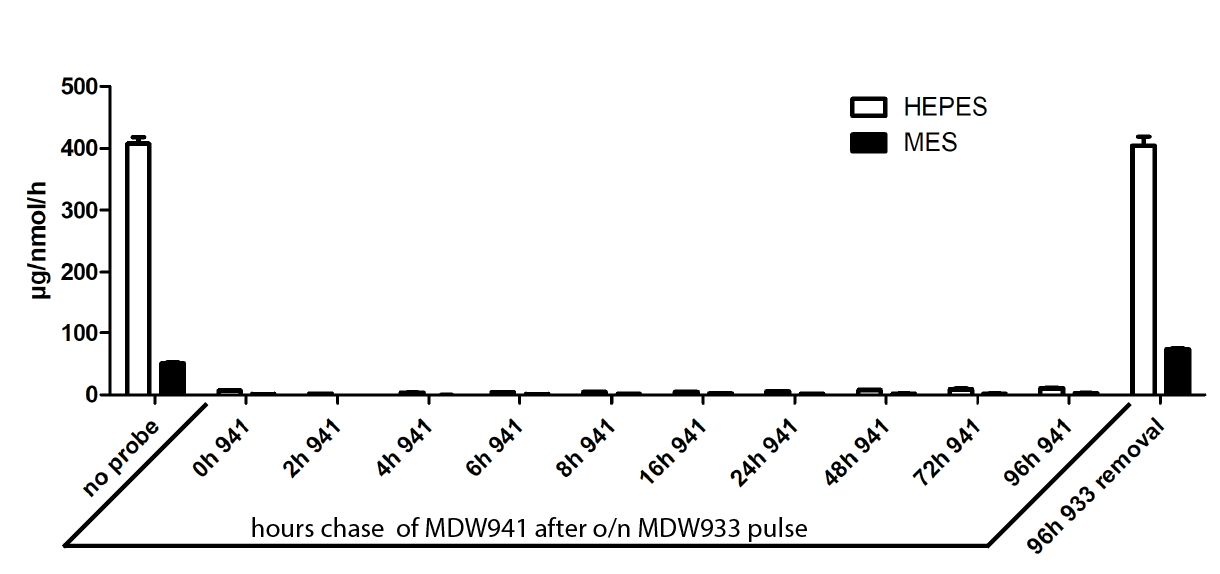


**Figure S2. Inhibition of GCase in pulse (MDW933)-chase (MDW941) experiments as measured by 4MU-assay.** Pulse-chase experiments with HEPES and MES treated RAW264.7 cells, performed as described in M&M. Following pre-labeling with MDW933 (green fluorescent), cells were incubated continuously with MDW941 (red fluorescent) for indicated time periods. Basal GCase activity was assessed in samples left untreated with probes. Recovery of GCase after block was assessed by applying pulse (MDW933) and subsequent culturing without probe for 96h. Cells were harvested and lysed for enzyme activity measurements using fluorogenic substrate for GCase as described in M&M.


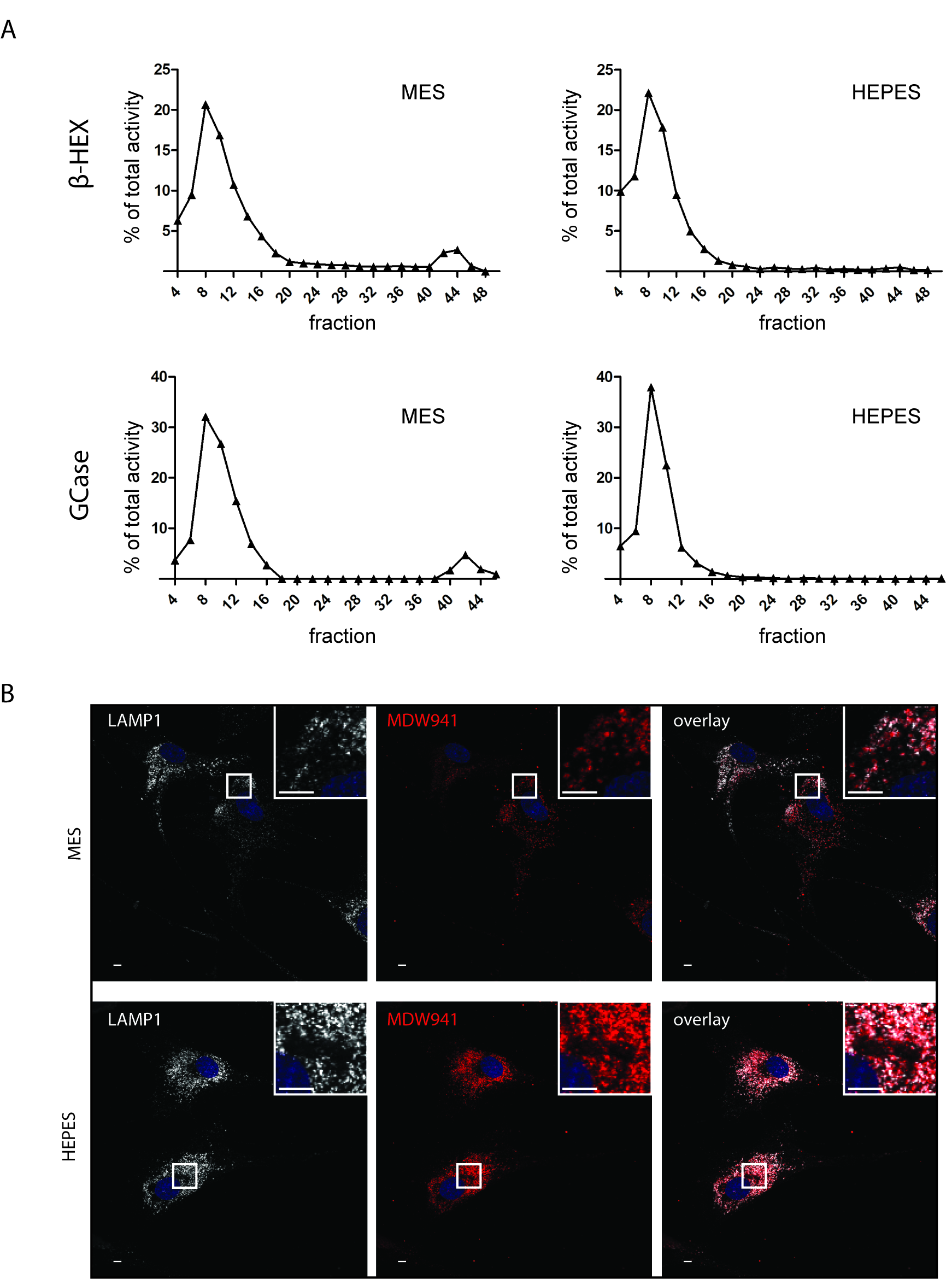


**Figure S3. Subcellular localization of GCase in the presence of 50 mM HEPES or MES. (A)** Normal human derived fibroblasts (NHDFs) were fractionated and compartments were separated on the basis of density using 49% Percoll centrifugation to generate density gradients. In collected fractions enzymatic activities of GCase and β-hexosaminidase were measured as described in M&M. **(B)** Immunofluorescence analysis on localization of ABP-labelled GCase (MDW941^24^) with lysosomal membrane protein LAMP1 in NHDFs cultured in the presence of 50mM MES or HEPES. Scale bar = 10 µm

**
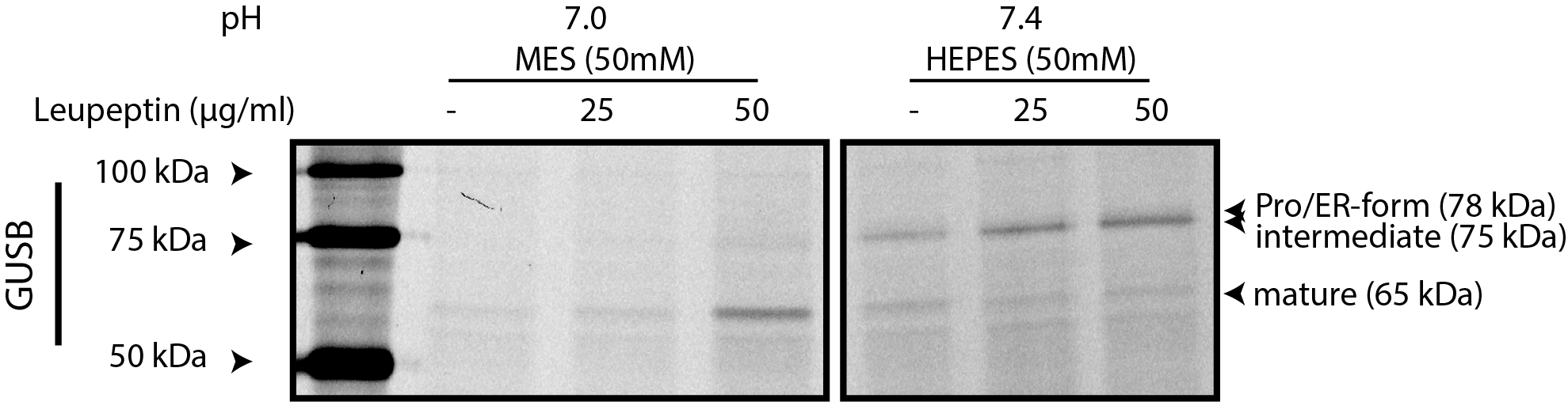
**

**Figure S4. Impact of HEPES on GUSB maturation in presence of leupeptin.** Fibroblasts were cultured in the presence of 50 mM buffer compound (MES or HEPES) simultaneously with 72h hour incubation with different concentrations leupeptin. Cells were harvested and GUSB in lysates was visualized by ABP labeling, SDS-PAGE and fluorescence scanning.

A
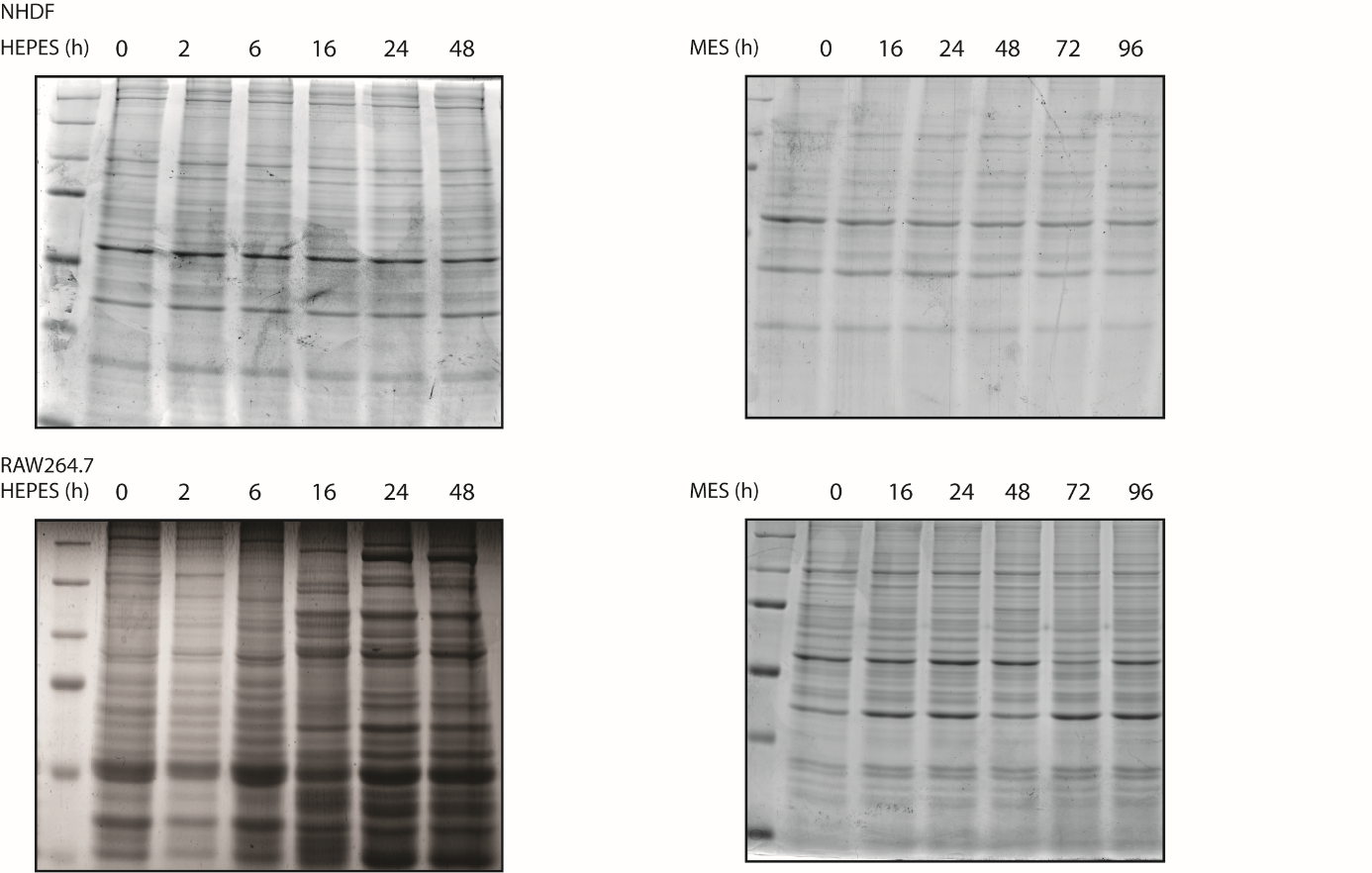


B


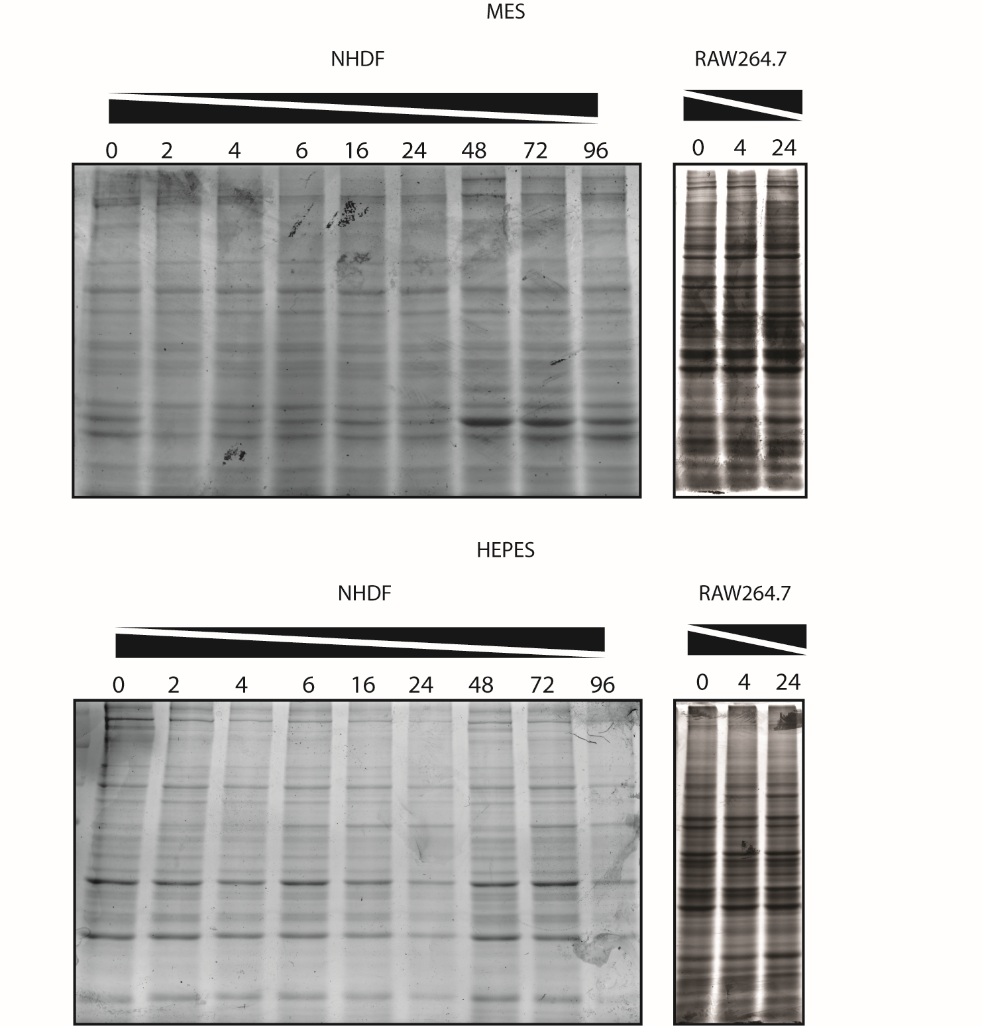


C


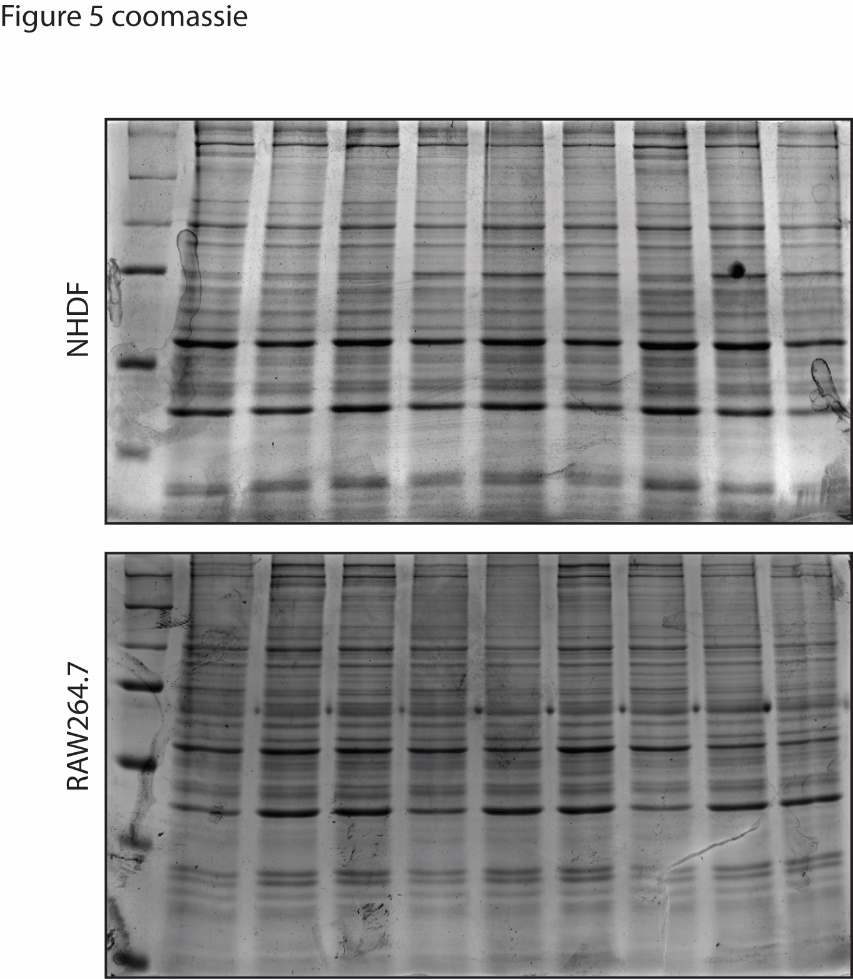


D


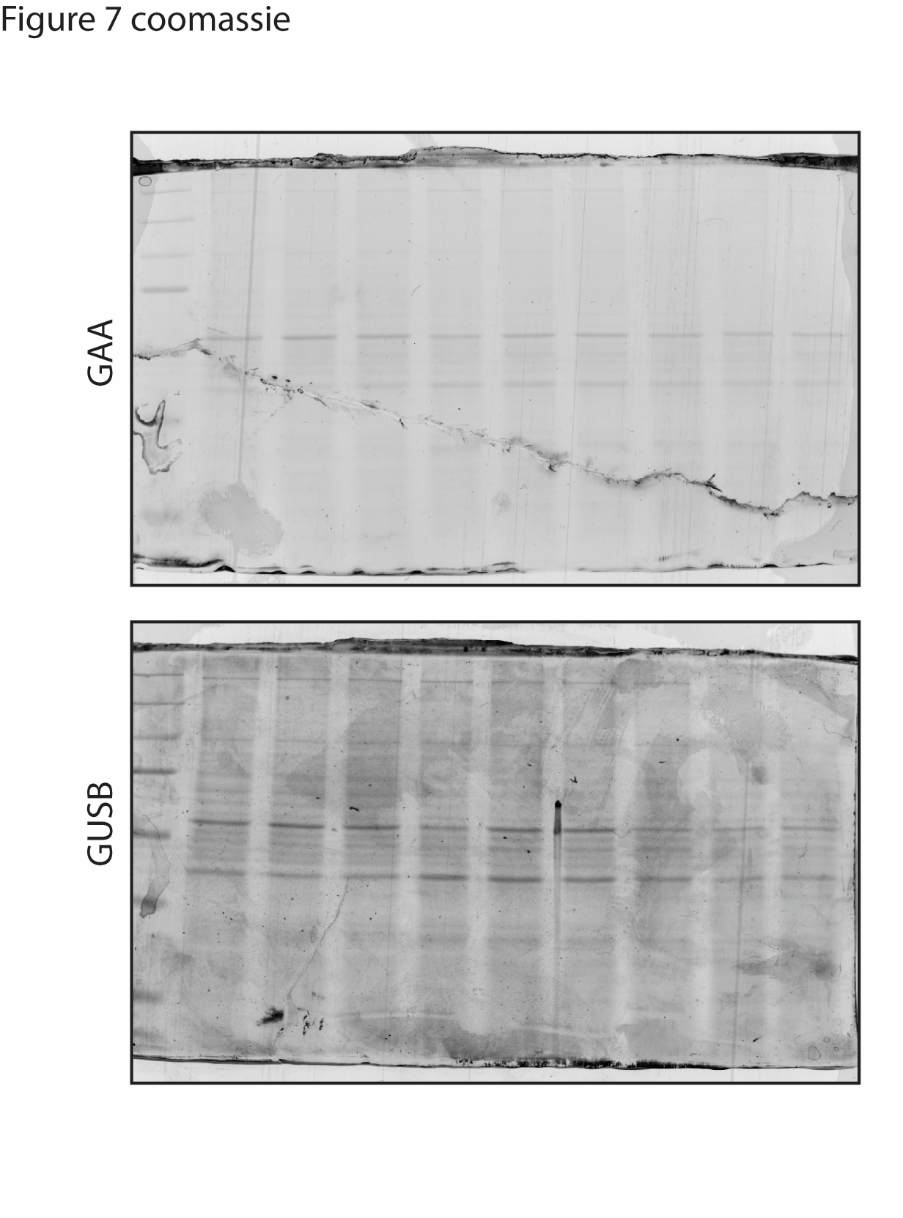


**Figure S5. Coomassie staining of gels depicted in Figure 2, 3, 5 and 7.** (A) Coomassie figures of Figure 2A (left) and 2B (right). (B) Coomassie figures of Figure 3A (left) and 3B (right). (C) Coomassie figures of Figure 5. (D) Coomassie figures of Figure 7.
